# The Effect of Protozoa on the Bacterial Composition and Hydrolytic Activity of the Roe Deer Rumen

**DOI:** 10.3390/ani10030467

**Published:** 2020-03-11

**Authors:** Renata Miltko, Barbara Kowalik, Małgorzata P. Majewska, Aneta Kędzierska, Neil R. McEwan, Grzegorz Bełżecki

**Affiliations:** 1 The Kielanowski Institute of Animal Physiology and Nutrition, Polish Academy of Sciences, 05-110 Jabłonna, Poland; b.kowalik@ifzz.pl (B.K.); m.majewska@ifzz.pl (M.P.M.); a.kedzierska@ifzz.pl (A.K.); 2 Robert Gordon University, School of Pharmacy and Life Sciences, Garthdee Campus. Robert Gordon University, Aberdeen AB10 7GJ, UK; n.mcewan@rgu.ac.uk

**Keywords:** bacteria, digestion, protozoa, rumen, wild roe deer

## Abstract

**Simple Summary:**

Ruminants are herbivorous animals which obtain their energy and nutrients from plant material due to a symbiotic relationship with microorganisms. The main site of this interaction is the rumen, where the intensive digestion processing of plant material takes place. The bacteria, fungi and protozoa create a unique ecosystem—a complex consortium based on mutual interactions. Protozoa are an important component of the microbiome. They have a major impact on the rumen ecosystem as well as on the ruminant’s welfare. Most information on protozoal importance in the digestive processes was obtained from studies on domestic ruminants, with results from studies on wild ruminants being limited. Therefore, the purpose of our study was to compare any effects of the presence of protozoa in the rumen of roe deer (*Capreolus capreolus*) on the bacterial composition and the digestion rate on the main carbohydrates of their forage. The results obtained from these pilot studies are presented as a short report investigating the relationship between these factors. The analysis of the bacterial composition indicated that the presence of protozoa did not have an effect on bacterial diversity, and furthermore, the protozoa had no effect on the digestion rate of carbohydrates in the rumen.

**Abstract:**

The purpose of this study was to compare the effect of the presence of protozoa in the rumen of wild roe deer (*Capreolus capreolus*) on the bacteria composition and digestion rate of the main carbohydrates of forage. The research material involved rumen content and rumen fluid, which were collected in the autumn-winter season, from eight adult males of roe deer with an average body mass of 22.6 kg. The microscopic analysis demonstrated that there were only protozoa in 50% of the animals sampled. Qualitative analysis revealed the presence of protozoa belonging to the genus *Entodinium*. The density of protozoal population varied from 6.5 to 38.7 × 10^5^/mL rumen fluid. The analysis of bacteria composition indicated that protozoa did not have an effect on bacterial diversity. Furthermore, the results of hydrolytic activity revealed that the fastest digestion of carbohydrates was for pectin, while the slowest was inulin. The pH and redox potential in the rumen varied from 5.9 to 6.1 and from −248.1 to −251.1 mV, respectively. In summary, the presence of protozoa in the rumen of wild roe deer does not have an effect on the bacterial population and has no effect on the digestion rate of carbohydrates in the rumen.

## 1. Introduction

Ruminants are herbivorous animals which obtain their energy and nutrients from plant material due to a symbiosis with microorganisms in their digestive tract. Ruminal microorganisms belong to three taxonomic groups: bacteria, fungi and protozoa. These microorganisms not only actively participate in the metabolism of all nutrients [1,2], but also thanks to the biochemical processes in their cells (e.g., synthesis of microbial proteins, incorporation of unsaturated fatty acids), they are a significant source of nutrients for ruminants [3]. The abundance, proportions and interactions between them are decisive factors in providing the needs of ruminants and for obtaining an appropriate quality and quantity of animal products for livestock production [4,5].

After bacteria, in terms of abundance and biomass content, protozoa are the second-largest group of microorganisms living in the rumen [6] and have a major impact on the rumen ecosystem of ruminants [6,7]. Moreover, in a generic context, the composition of ruminal protozoa depends on both the host species as well as the content of its diet. For example, it is different in an animal fed with concentrated feed relative to one on a more cellulose-rich diet [8]. Furthermore, the presence of protozoa in the rumen has been shown to be pH-dependent; i.e., protozoa belonging to the genus *Entodinium* were established at a pH slightly above 6.0, while other protozoa did not develop until the pH reached 6.5 or above [8]. Another study demonstrated that the population of protozoa was different in different seasons and periods of mammalian development. The infection of mammals with their specific microfauna can occur by several means; e.g., by the licking and transfer of saliva from the mother to her young, as well as the transfer of protozoa inoculum between animals by salivation on the feed or pasture. However, their role in this ecosystem and their digestive capacity are still poorly understood. Although not complete, some information is available. For example, one of the well-documented effects of protozoa is their ability to modify the composition of the rumen bacterial population [9] and rumen fungal population [10]. Thus, they can indirectly have an effect on fibrolytic activity [11] or starch [12] and chitin degradation [13].

Most of the information on mutual protozoa–bacteria interactions has been derived from domesticated livestock, classified according to their food preferences by Hoffman [14] into grazer ruminants (cattle and sheep) or intermediate feeder ruminants (goats). However, the current literature lacks information about mutual microbial interactions and their influence on rumen digestive activity in wild ruminants.

Roe deer (*Capreolus capreolus*) are the most abundant cervids in Europe [15] with great economic importance, both in terms of meat production and sport hunting. *C. capreolus* also have an ecological value as part of European biodiversity [16]. Roe deer are herbivores and belong to the group of ruminants of the “browsers” type; i.e., they are selective. In other words, they have a highly developed food selectivity, with a preference for those with significant nutritional values [14]. Considering their type of feeding, roe deer have a relatively small rumen, fast digestive flow and relatively large salivary glands [14]. Another unique feature observed in this species is the diversity of the population, which consists of individuals with developed populations of rumen protozoa (with protozoa) as well as those lacking them (without protozoa) [17]. The presence of protozoa can be an influencing factor impacting bacterial diversity and thus has an effect on rumen hydrolytic activity. Wild roe deer prefer easily digestible parts of plants and require food with a minimum digestibility of 58%. Herbaceous plants predominate in their spring and summer diet, with the young leaves, buds, green shoots of trees and shrubs also being of great importance. The winter diet of the wild roe deer is of poor quality and is dominated by shoots of trees and shrubs, dry grasses, herbs and lichen [18]. For these reasons, it is necessary to feed roe deer in the winter. Excessive deer populations may cause serious damage to agricultural crops and forest plantations as they try to source food. It is also worth emphasizing that, recently, a large number of countries in Europe, such as Germany, Switzerland, Austria, Sweden and Poland, have been developing increasing numbers of breeding programmes for cervid animals for meat production. Therefore, all information regarding nutrition and digestive processes in these animals can contribute to the better management of their population in the wild and in breeding for agricultural purposes.

Considering all of the above, the aim of the present study was to examine the effect of the presence of rumen protozoa on bacteria populations and the hydrolytic enzymatic activity of the rumen ecosystem.

## 2. Materials and Methods

### 2.1. Animal and Sample Collection

The material for the study was collected during the 2011/2012 hunting season, between November and January from the Ciechanów Forest district in North Poland (52°56′08′′ N, 20°29′58′′ E) as part of the planned hunting program. The research material involved collections of rumen content and rumen fluid, which were collected from eight adult (2–3 years old) male roe deer (*Capreolus capreolus*) with an average body mass of 22.6 kg. The age of a culled animal was determined by inspecting the dentine layers [19]. Immediately after killing each individual deer, its rumen was emptied and the contents (approximately 2.5 kg) were mixed and divided into two parts. The first portion (5 g) was frozen and stored (−80 °C) to test bacterial diversity, and the second portion (20 g) was frozen and stored (−20 °C) for hydrolytic activity analysis. To obtain a representative sample, rumen fluid was collected from the dorsal and ventral sacs of the rumen and thoroughly mixed and filtered through four layers of gauze (1 mm pore size). The pH and the redox potential (Eh) were measured immediately with a pH meter (model 7011, ChemLand, Poland) equipped with respective electrodes. The dry weight of the rumen content was determined by heating the content at 105 °C for 48 h and expressing this value as a percentage of the original wet weight of the material.

An aliquot of the filtrate (5 mL) was fixed in a 4% formalin solution (5 mL) for subsequent protozoa analysis. A preliminary microscopic analysis of the fixed rumen fluid indicated the presence of protozoa in four of the eight animals examined. This allowed a comparison of the effect of protozoa on hydrolytic activity in the four individuals with protozoa versus the four individuals without protozoa, as well as a comparison of their bacterial diversity.

### 2.2. Determination of Protozoa Numbers

Protozoa in the formalin solution were identified based on morphological criteria, according to Dogiel [20], which were as follows: presence of skeletal plates; the number and size of ciliary zones; the number and location of contractile vacuoles; the size, position and shape of the nuclear apparatus (macro- and micronucleus); the number of spines/lobes; overall body size; and the shape of the cell. The samples were treated with carmine, chrome alum (Fyg), dyeing the nuclei dark blue and the skeletal plates were stained with Lugol’s iodine. After 14 days of dyeing, qualitative and quantitative analysis of the ciliate populations were performed with a fluorescent microscope Olympus DP 50 (magnification ×100, Olympus, Tokyo, Japan) as described previously by Miltko et al. [21].

### 2.3. Bacterial Diversity Analysis

#### 2.3.1. DNA Isolation

For the determination of bacterial diversity, the total genomic DNA was isolated from the rumen fluid using a ZR Fecal DNA Kit (Zymo Research, Irvine, CA, USA) according to the manufacturer’s instructions. The amount and purity of the extracted DNA was tested by UV spectrophotometric analysis at 230, 260 and 280 nm using a NanoDrop ND-1000 spectrophotometer (Thermo Fisher Scientific Inc., NanoDrop Technologies, Inc., Wilmington, DE, USA).

#### 2.3.2. PCR Amplification

PCR was performed using a bacterial-specific primer pair, cyanine-labelled 27F (5’-AGA GTT TGA TCC TGG CTG AG-3’) and unlabeled 1389R (5’-AGG GGG GGT GTG TAG AAG-3’), [22]. Amplification was performed using a BIORAD MyCycler™ thermal cycler (BIORAD Laboratories, Inc, Hercules, CA, USA) and the following program: initial 4 min of denaturation at 94 °C, then 25 cycles of 1 min denaturation at 94 °C, 1 min annealing at 55 °C and 1 min extension at 72 °C. The final cycle of 1 min at 94 °C, 1 min at 55 °C and elongation for 5 min at 72 °C completed the reaction. The reaction cocktail contained: 0.05 U/µL Taq DNA polymerase (Promega, Madison, WI, USA); 1× reaction buffer as supplied by the manufacturer; 1.5 mM MgCl_2_; 0.25 µM of each primer; and 0.2 mM of each dNTP with 2 µL of DNA template. All reactions were carried out in a final volume of 50 µL.

#### 2.3.3. Restriction Enzyme Digestion of Amplicons

PCR products were purified on a Millipore MultiScreen^®^ PCR plate (with 20 inches Hg vacuum) (Merck Millipore Burlington, MA, USA). The DNA concentration for each sample was determined spectrophotometrically using a Nanodrop^®^ ND-1000 spectrophotometer (Thermo Fisher Scientific Inc., NanoDrop Technologies, Inc., Wilmington, DE, USA) and subsequently diluted to 20 ng/µL. Restriction enzyme digestion was performed using HhaI and MspI (Promega) in single enzyme digestions at 0.15 U/µL at 37 °C for 5 h. The purified restriction digestion product (20 µL) was added to 1 µL of DNA size standard 600 (diluted 1:3 with SLS) and 20 µL of SLS (Beckman Coulter Inc., Fullerton, USA). Terminal restriction fragments (TRFs) were determined using the local southern algorithm, calibrated using an AE version 2 (manufacturer’s recommendation, CEQ™ 8000 Beckman Coulter software, (285501, AB SCIEX. SCIEX, Framingham, MA, USA) and aligned using the AFLP (amplified fragment length polymorphism) functionality in the CEQ™ 8000 software.

#### 2.3.4. Exclusion Parameters for Analysis of Terminal Restriction Fragments (TRFs)

Exclusion parameters were applied, whereby all TRFs that accounted for less than 1% of the total area under the curve (AUC) were excluded [23].

#### 2.3.5. Analysis of Similarity Levels between TRF Patterns

Analysis of TRF samples was performed using Manhattan distances to generate a distance matrix. Subsequently, UPGMA (unweighted pair group method with arithmetic mean) analysis was performed using Neighbor included in the PHYLIP software suite [24].

### 2.4. Measurement of Hydrolytic Activity

In order to determine the hydrolytic activity of rumen samples, an enzymatic fraction was extracted using the carbon tetrachloride and lysozyme method as described by Miltko et al. [25]. The resulting enzymatic fraction was used to determine the digestion rate of storage compounds (starch and inulin) and structural carbohydrates (xylan, cellulose and pectin), according to Miltko et al. [25]. The reaction mixture contained substrate (solutions or suspensions of one of the following carbohydrates: starch, inulin, xylan, carboxymethyl cellulose or pectin); enzymatic fraction; and 0.01 M phosphate buffer (pH 6.0). The mixtures were incubated for 1 h at 40 °C and the reaction was stopped by the addition of dinitrosalicylic reagent, followed by spectrophotometric absorbance measurements at 560 nm. The concentration of reducing sugars released from the substrates was calculated by comparing the absorbance of hydrolysis end products for the respective substrates, and these were expressed as equivalents of monosaccharides per g dry matter (DM) rumen contents per unit of time.

### 2.5. Statistical Analysis

The measurements were performed in triplicate for each sample. The results were presented as mean value ± standard deviation (SD) as well as maximum and minimum values. Data for total protozoal concentration and hydrolytic activity were statistically analyzed by a one-way analysis of variance (ANOVA) to compare between roe deer with protozoa and roe deer without protozoa. The differences were considered significant at *p* ≤ 0.05, whereas in the *p* ˃ 0.05 and *p* < 0.10 range were defined as trends. Statistical analysis was performed using Statistica program (StatSoft Inc. Tulsa, OK, USA).

## 3. Results

The microscopic analysis demonstrated that there were protozoa in the rumen of 50% of the animals studied. Qualitative analysis of the rumen fluid revealed the presence of protozoa belonging to the *Ophryoscolecidae* family, represented by species of the genus *Entodinium* (*E. caudatum*, *E. simplex*, *E. monolobum*, *E. dilobum*, *E. longinucleatum*, *E. minimum*, and *E. rostratum*). The density of ciliate populations varied from 6.5 × 10^5^ to 38.7× 10^5^/ mL rumen fluid.

**Figure 1:**
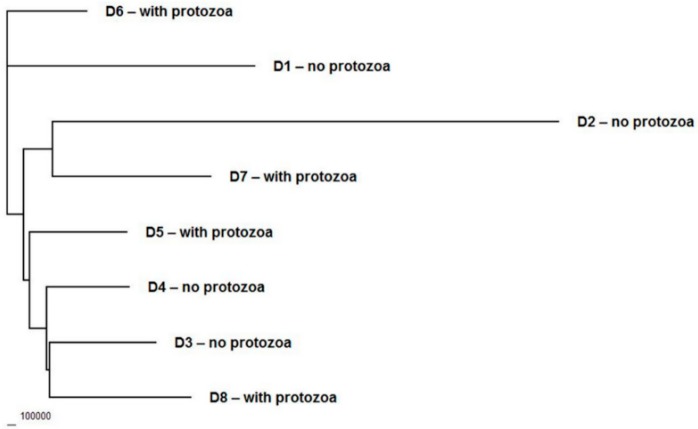
Dendrogram depicting the relationship of bacterial diversity between samples from roe deer (*Caprelous caprelous*) with rumen protozoa (D1–D4) or without protozoa (D5–D8).

The differences obtained created the opportunity to undertake a study on the impact of protozoa on the bacterial population. The analysis of the bacterial diversity indicated that the protozoa had no detectable effect on the bacterial population (Figure 1). The measurement of hydrolytic activities (Table 1) showed that the presence of protozoa only increased the starch degradation rate by 30%. However, due to the range of values across both groups of animals, as with other enzymatic values, this result was not significantly different (*p* > 0.05), nor was it different enough to be considered as a trend (*p* > 0.10).

The presence of protozoa in the rumen of roe deer did not have an effect on the value of the pH and redox potential, which ranged from 5.9 to 6.2, and from −248.1 to −251.1 mV, respectively.

## 4. Discussion

The current study found that only 50% of the roe deer examined had rumen protozoal fauna. The finding that not all roe deer carry ciliated protozoa is consistent with previous results. For example, Marinucci et al. [17] did not find any protozoa in the individual animals examined; in contrast, Drescher-Kaden and Seifelnasr [26], Enzinger and Hartfiel [27] and Kamler [28] observed protozoa in all animals examined. The concentration of protozoa observed in the present study was higher than that of roe deer examined by Kamler [28], but lower than in the studies of Enzinger and Hartfiel [27]. Variation in the microfauna composition between animals was detected. This was the case in the present study as well as in the works of Drescher-Kaden and Seifelnasr [26], Deutch et al. [29] and Marinucci et al. [17]. Here, only a single protozoal genus was detected; i.e., *Entodinium*. In contrast, Enzinger and Hartfiel [27] also found members of the genera *Diplodinium, Epidinium, Polyplaston* and *Ophryoscolex,* but in low numbers. Several factors could be responsible for such diversity, one of which could be the dietary factor. In the experimental study in [27], feeding roe deer a roughage diet supplemented with low and high concentrate feeds increased the total number of protozoa observed—specifically *Entodinium* spp.—but at the same time, a complete disappearance of other protozoal species was observed. These results could explain the increase in the total concentration of protozoa in roe deer in the summer [28] when the diet is richer in easily digestible carbohydrates relative to winter [18], but they could not explain the disappearance of other genera of ciliates. A decrease in the pH value to 5.9 has been observed previously [27], but this is not sufficient to eliminate protozoa, because they are able to survive to pH values as low as 5.3 [30].

The second and probably the most important factor is the feeding type of animals. Roe deer belong to concentrate selector ruminants and are characterized by their short ruminal retention times [31]. Protozoa that require a longer time for cell division could easily be washed out of the rumen. Generation times differ between species; for example, they are shorter in *Entodinium exiguum* than in larger ciliates, such as *Eudiplodinium maggii* and *Ophryoscolex purkynjei* [32], which have a longer doubling time. This could be the reason for the changes in the protozoal population observed by Enzinger and Hartfiel [27] or even their absence, as observed in some animals in the current study. The absence of protozoa in the rumen could be a permanent feature of this species due to the solitary nature of the roe deer, and the lack of contact with other animals might have prevented the trans-faunation and building or rebuilding of a stable protozoal population [33].

Irrespective of the reason for roe deer non-faunation in some animals, we hypothesized that the presence of protozoa would affect the bacterial population, as previously observed in domesticated ruminants [9]. However, such a phenomenon was not detected in this study (Figure 1). The reasons for this could relate to the material obtained from the wild living population foraging in different areas or to the solitary nature of these animals. Perhaps the effect of the individual’s diet outweighs the influence of the presence of protozoa. Future research needs to be conducted on larger groups of animals to resolve this issue. It is possible that there are differences in low-abundance sequences which might not be detected by the method used here. However, there is evidence to suggest that, relative to next generation sequencing, Terminal Restriction Fragment Length Polymorphism (T-RFLP) is just as effective at picking up population differences at the microbiome level as next-generation approaches [34], except that the identity of the differences is not determined. Therefore, we are confident that the data presented here are a true representation of the inter-individual bacterial population comparisons. However, even with different organisms present, it is possible that similar metabolic functions are carried out by those bacteria present in the rumen. With the exception of a single article focused on cellulolytic activity [29], the existing literature lacks information on the degradation parameters of food carbohydrates for roe deer. The methods applied here for measuring hydrolytic activities were those previously employed in our studies on sheep [25]. A comparison between these animals indicated that sheep digested all carbohydrates to a higher extent, with the exception of pectin, which was digested at a comparable level. This was similar to the results obtained by Enzinger and Hartfiel [27], who compared short chain fatty acid concentrations in roe deer and goats and also found a lower microbial metabolic activity in roe deer. Comparing the degradation rate of particular substrates, it was found that amylolytic activity was three times higher in sheep. However, these animals were fed a diet rich in barley, whereas roe deer were foraging on low-energy winter food. Cellulose and xylan were digested by roe deer at a low rate despite the fact that carbohydrate-rich plants are their main food component in winter [29]. These results could be explained by food adaptation of these animals. According to Hofmann [14], the classification of ruminants represents two different feeding types: concentrate selectors-browsers (e.g., roe deer) or roughage feeders-grazers (e.g., sheep). The concentrate selectors are adapted to a faster-fermenting diet [35] and have a less efficient cellulose digestion compared to grazers [14].

The presence of protozoa did not cause significant changes (*p* > 0.05) in the enzymatic activities studied; however, we observed a small but not significant increase of amylolytic activity in roe deer with protozoa, which could be due the presence of *Entodinium* and their ability to metabolize starch [12]. To address this hypothesis, studies on a larger group of animals will be undertaken in the future.

Moreover, in the present study, we did not observe increased cellulolytic activity, as previously reported by Deutch et al. [29]. This may be due to a difference in dietary composition or geographical location between these studies or the lower number of animals examined in the current study. Future investigations on larger groups of animals may help to resolve this issue.

In summary, the presence of protozoa in the roe deer rumen does not affect the bacterial population to a greater extent than the difference in the composition of microflora naturally occurring between other animals (intra-individual changes). Moreover, in our study, the protozoa did not affect the hydrolytic activity of rumen contents, and therefore it can be assumed that it also did not have an effect on the digestive processes in the rumen.

## Figures and Tables

**Table 1:** Comparison of protozoa numbers (×10^5^/mL rumen fluid) and hydrolytic activities (µM of released monosaccharide/g dry matter of content per minute) in the rumen of roe deer (*Caprelous caprelous*) with protozoa and without protozoa.

Item	Roe Deer with Protozoa				Roe Deer without Protozoa			
	Min	Max	Mean	SD	Min	Max	Mean	SD
Total protozoa (*Entodinium* spp.) ^1^	6.50	38.70	16.45	15.17	-	-	-	-
Amylolytic activity	1.41	3.69	2.42	0.95	1.01	2.30	1.86	0.58
Celulolytic activity	2.54	2.92	2.66	0.17	1.61	3.72	2.60	0.88
Xylanolytic activity	3.00	4.28	3.47	0.59	3.27	4.46	3.85	0.58
Inulinolytic activity	1.05	2.09	1.49	0.43	1.23	1.77	1.38	0.26
Pectinolytic activity	3.49	4.43	3.92	0.50	2.85	4.06	3.76	0.65

Values shown are minimum, maximum, mean and standard deviation. No significant differences (*p* > 0.05) or trends towards a difference (*p* > 0.10) were observed between roe deer with protozoa and without protozoa.^1^ (×10^5^/ mL rumen fluid).
